# Simulation analysis of impact damage to the bone tissue surrounding a dental implant

**DOI:** 10.1038/s41598-020-63666-5

**Published:** 2020-04-24

**Authors:** Xinyang Ma, Xiaoou Diao, Zhirui Li, Haitao Xin, Tao Suo, Bing Hou, Zhongbin Tang, Yulu Wu, Fan Feng, Huiwen Luo

**Affiliations:** 1 State Key Laboratory of Military Stomatology & National Clinical Research Center for Oral Diseases & Shaanxi Key Laboratory of Stomatology, Department of Prosthodontics, School of Stomatology, The Fourth Military Medical University, Xi’an, 710032 China; 2 School of Stomatology, XI’AN Medical University, Xi’an, 710021 China; 3 School of Aeronautics, Northwestern Polytechnical University, Xi’an, 710072 China

**Keywords:** Computational models, Experimental models of disease, Computational science

## Abstract

Dental implant may suffer transient external impacts. To simulate the effect of impact forces on bone damage is very important for evaluation of damage and guiding treatment in clinics. In this study, an animal model was established by inserting an implant into the femoral condyle of New Zealand rabbit. Implant with good osseointegration was loaded with impact force. A three-dimensional finite element model was established based on the data of the animal model. Damage process to bone tissue was simulated with Abaqus 6.13 software combining dynamic mechanical properties of the femur. The characteristics of bone damage were analyzed by comparing the results of animal testing with numerical simulation data. After impact, cortical bone around the implant and trabecular at the bottom of the implant were prone to damage. The degree of damage correlated with the direction of loading and the magnitude of the impact. Lateral loading was most likely performed to damage cancellous bone. The stress wave formed by the impact force can damage the implant–bone interface and peri-implant trabeculae. The data from numerical simulations were consistent with data from animal experiments, highlighting the importance of a thorough examination and evaluation based on the patient’s medical history.

## Introduction

With the development of implant materials and technology, the use of dental implants to replace lost teeth has become increasingly popular. Alveolar bone plays a vital role in the stability of dental implants throughout the process of osseointegration. Therefore, good osseointegration is key to the success of dental implants^1,2^. Normal masticatory and physiological forces can improve the remodeling of alveolar bone, thus maintaining the stability of bone tissue surrounding the implant^3,4^.

However, in emergency situations such as traffic accidents, sports-related injuries, soldier training, geological disasters, and military conflicts, the dentition and dental implant may be subjected to transient external impacts. In natural teeth, the periodontal ligament acts as a cushion to buffer the force of the impact. There is no periodontium surrounding dental implants, therefore the impact force acts directly on the alveolar bone and propagates sometimes in the form of stress waves through the composite structure consisting of the implant and bone. Alveolar bone can buffer and absorb the impact energy, resulting in structural and morphological changes^5,6^. When the force of the impact exceeds the capacity of the alveolar bone to sustain its structural and morphological integrity, the implant–bone interface may be damaged. Microstructural changes in surrounding bone tissue^7^ may destroy the osseointegration, resulting in a loss of implant stability and ultimately in implant failure. Few studies have investigated the damage to bone tissue associated with a forceful blow to implants. No previous numerical study has been performed to simulate the process by which bone surrounding the implant is damaged during a forceful blow.

In the present study, implants were placed in the femoral condyles of New Zealand rabbits. All implants with good osseointegration were subsequently loaded an impact force. A three-dimensional (3D) finite element (FE) model (including implant, cortical bone and cancellous bone) was established based on the animal model. In combination with data from dynamic mechanical experiments of impact loading on femur, this FE model was used to simulate the process and characteristics of bone damage in the area surrounding the implant. The damage caused by impact and the propagation of stress waves in the bone surrounding the implant was investigated under various impact loads. This study aims to clarify the association of impact load with bone damage and to elucidate the mechanism underlying impact-related damage. The information presented may provide a theoretical basis for the evaluation of damage and guide clinicians in planning treatment for patients with impacted implants.

## Materials and methods

### Animal experiments

#### Implant implantation

**Figure 1:**
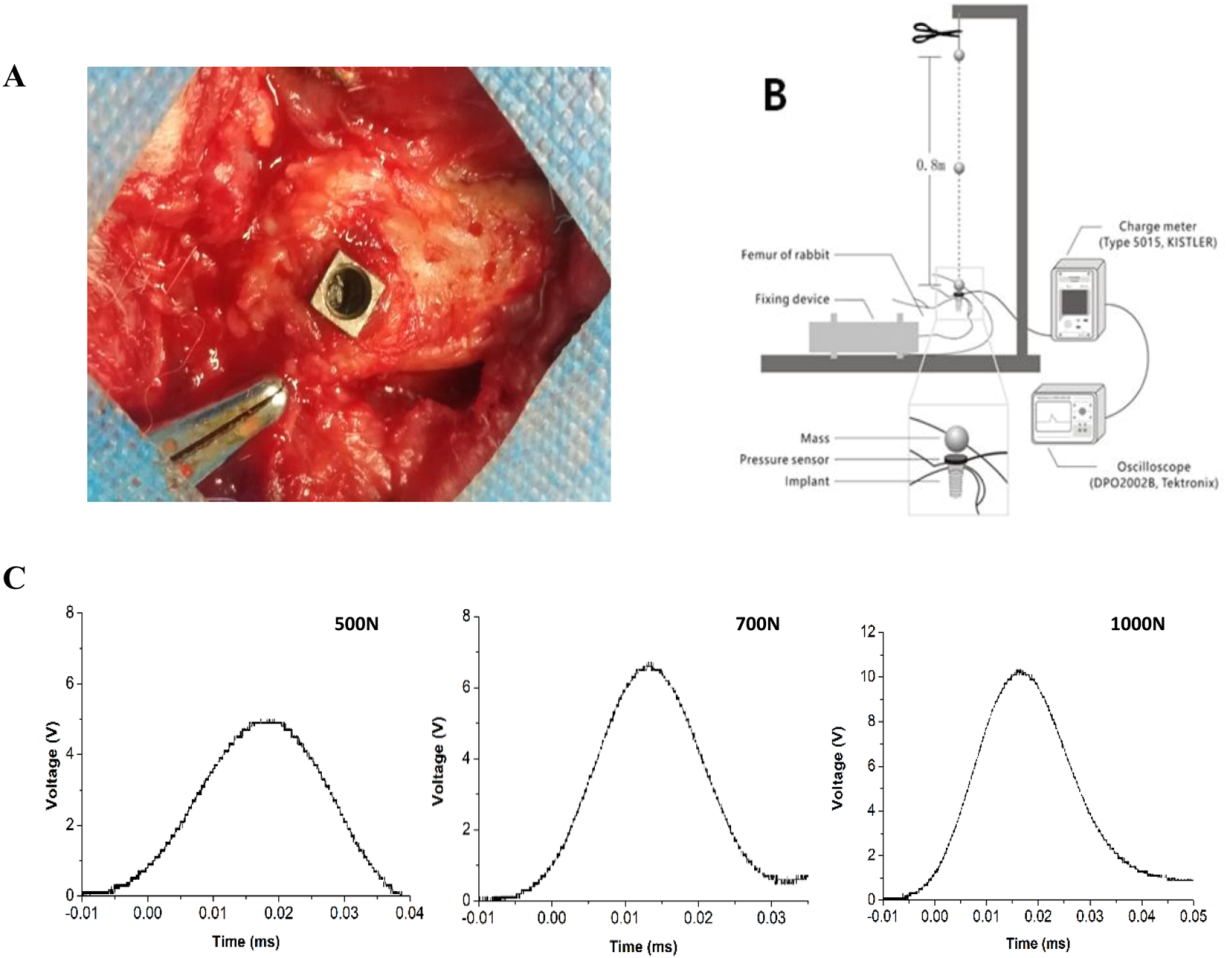
Implantation surgery and impact loading. (**A**) The distal femoral condyle was chosen as the site for implantation. (**B**) Schematic of impact loading. (**C**) Voltage values obtained under various rates of impact loading.

Twenty-four New Zealand white rabbits (weight ~2.5 kg) were chosen for this study. Eighteen received implants designed and manufactured using commercially pure titanium (CP Ti) in our laboratory (diameter, 4 mm; length, 8 mm;) (Fig. 1A). The six animals without implants were used for dynamic mechanical testing. The implants were inserted into the bilateral femoral condyle of rabbit, as described previously^8^. All surgical procedures were performed painlessly under general anesthesia using 1 mL/kg 3% pentobarbital sodium and 0.1–0.2 mL/kg 846 mixture by intramuscular injection. After three months, osseointegration was analyzed by micro-computed tomography (Micro-CT) scan and histomorphometry.

After osseointegration, animals with implants were randomly divided into 3 groups according to impact load (n = 6 in each group). In each group, one side of the femoral condyle was assigned to the experimental group randomly; the other was assigned to the control group.

#### Impact loading

In the test group, animals were subjected to impact loading with the drop-hammer method. During loading, a 15-g solid steel ball was dropped from a height of 0.8 m, 1.5 m, and 2.0 m (Fig. 1B) onto a pressure sensor (9001A, KISTLER, Switzerland) connected with the implant. The pressure was received and magnified by the charge meter (Type 5015, KISTLER, Switzerland) as voltage signal. The voltage value was recorded (Fig. 1C) and converted to loading force (500N, 700N, 1000N) correspondingly. Once it had contacted the implant surface, the solid ball was quickly removed. All animals were sacrificed using over-dose anesthesia immediately after impact loading. Bone tissue in the 1-cm margin surrounding the implant site was collected. Micro-CT and histomorphometric analysis were subsequently performed.

The study was conducted at the Animal Center of the Air Force Military Medical University, Xi’an, China, according to the institution’s guidelines for the care and use of laboratory animals. The protocol was approved by the Laboratory Animal Protection and Welfare Committee of the Air Force Military Medical University, Xi’an, China (No. 2018-007).

#### Micro-CT and histomorphometric analysis

The implants with surrounding bones were cut out and fixed in 4% paraformaldehyde. The specimens were scanned using micro-CT (Inveon Research Workplace 2.2, Siemens, Germany) at a resolution of 39.28 µm, with 80 kV voltage and 50 uA current. The bone-implant structure was reconstructed and analysed by the Inveon Acquisition Workplace 2.2 scanner program (Siemens, Germany). A 0.5-mm area around the implant was chosen as a region of interest (ROI) for longitudinal analysis of the peri-implant bone microstructure. Parameters related to bone volume/total volume (BV/TV, %) and trabecular separation (Tb.Sp, mm) were calculated to evaluate osseointegration and the extent of bone damage after impact loading.

After evaluation with Micro-CT, samples were prepared for histological study. The samples were dehydrated in a graded alcohol series, embedded in methyl methacrylate, and cut into longitudinal sections of 200-µm thickness. The sections were ground to 20 µm and stained with Van Gieson (VG). The images were captured with a microscope (DMI6000, Leica, Germany) and the bone-to-implant contact (BIC) was calculated to evaluate the extent of osseointegration and damage. Micro-CT and histological data were used for subsequent verification of the numerical simulation analysis.

#### Dynamic mechanical testing of femoral cortical and cancellous bone

In order to simulate the impact process, rabbit femurs were submitted to dynamic mechanical testing.

Femurs that had not received implants were embedded and fixed with one side on the low-speed cutting machine (SYJ-150, YLSHD, Beijing, China). Specimens of cancellous (5 × 6 × 6 mm) and cortical bone (6 × 3 × 8 mm) in different section of femur were cut respectively.

**Figure 2:**
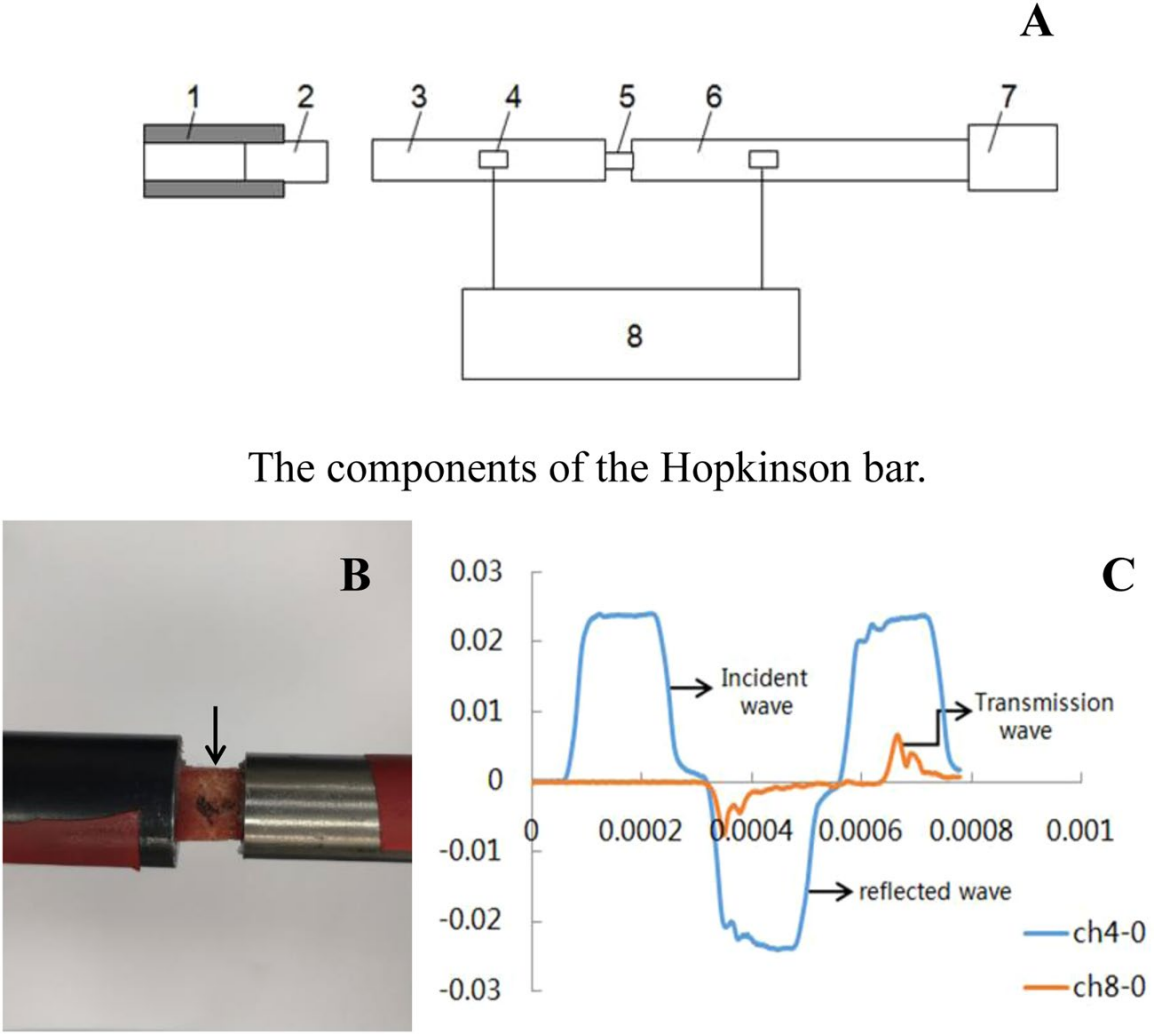
Schematic of Hopkinson bar and dynamic mechanical testing of rabbit femur. (**A**) Description of the Hopkinson bar. 1. Gas gun; 2. projectile; 3. Input bar; 4. strain gauge; 5. test sample; 6. output bar; 7. energy-absorbing device; 8. data acquisition and processing system (**B**) Fixation of the specimen on the Hopkinson bar (black arrow). (**C**) Incident wave, reflected wave, and transmitted wave, as recorded by the strain gauge.

Dynamic compression tests of cortical and cancellous bone were performed on the Split Hopkinson Pressure Bar (SHPB)^9,10^, which consists of a projectile, an input bar, and an output bar (Fig. 2A). All bars are made of steel, with density of 7830 kg/m^3^ and Young’s modulus of approximately 209 GPa.

The bone specimen was then sandwiched between the input and output bars (Fig. 2B). A projectile, launched by a gas gun, struck the free end of the output bar and initiated a compressive longitudinal incident wave. Once this wave reached the bar/specimen interface, part of it was reflected, whereas the other part traveled through the specimen and developed the transmitted wave in the output bar. To record these three basic wave types, two strain gauge pairs were cemented at the midpoints of the input and output bars (Fig. 2C). The stress and strain rate, as well as strain histories, can be calculated from those three basic waves according to the one-dimensional stress wave theory^11,12^, as follows:

$${\sigma }_{s}=E\left(\frac{{A}_{b}}{{A}_{s}}\right){\varepsilon }_{T}$$

$${\varepsilon }_{s}=-\left(\frac{2{C}_{0}}{{l}_{s}}\right){\int }_{0}^{t}\,{\varepsilon }_{R}dt$$

$${\dot{\varepsilon }}_{s}=-\left(\frac{2{C}_{0}}{{l}_{s}}\right){\varepsilon }_{R}$$

where ${\sigma }_{s}$ is the stress of the specimen; $E$ is Young’s modulus of the bars; As is cross-sectional area of the bar; ${A}_{s}$ is cross-sectional area of the specimen; ${\varepsilon }_{T}$ is the transmitted wave; ${\varepsilon }_{s}\,$is strain of the specimen; ${C}_{0}$ is wave velocity of the incident bar; ${l}_{s}\,$is length of the specimen before deformation; ${\varepsilon }_{R}$ is the reflected wave; ${\dot{\varepsilon }}_{s}$ is the strain rate of the specimen.

In these SHPB tests, the impact velocity of the projectile was approximately 10 m/s, which ensured that the strain rates of the bone specimen were in the same order of magnitude with that of the animal experiments.

### Numerical simulation model of the implant and surrounding bone

#### reconstruction of the implant and bone tissue

**Figure 3:**
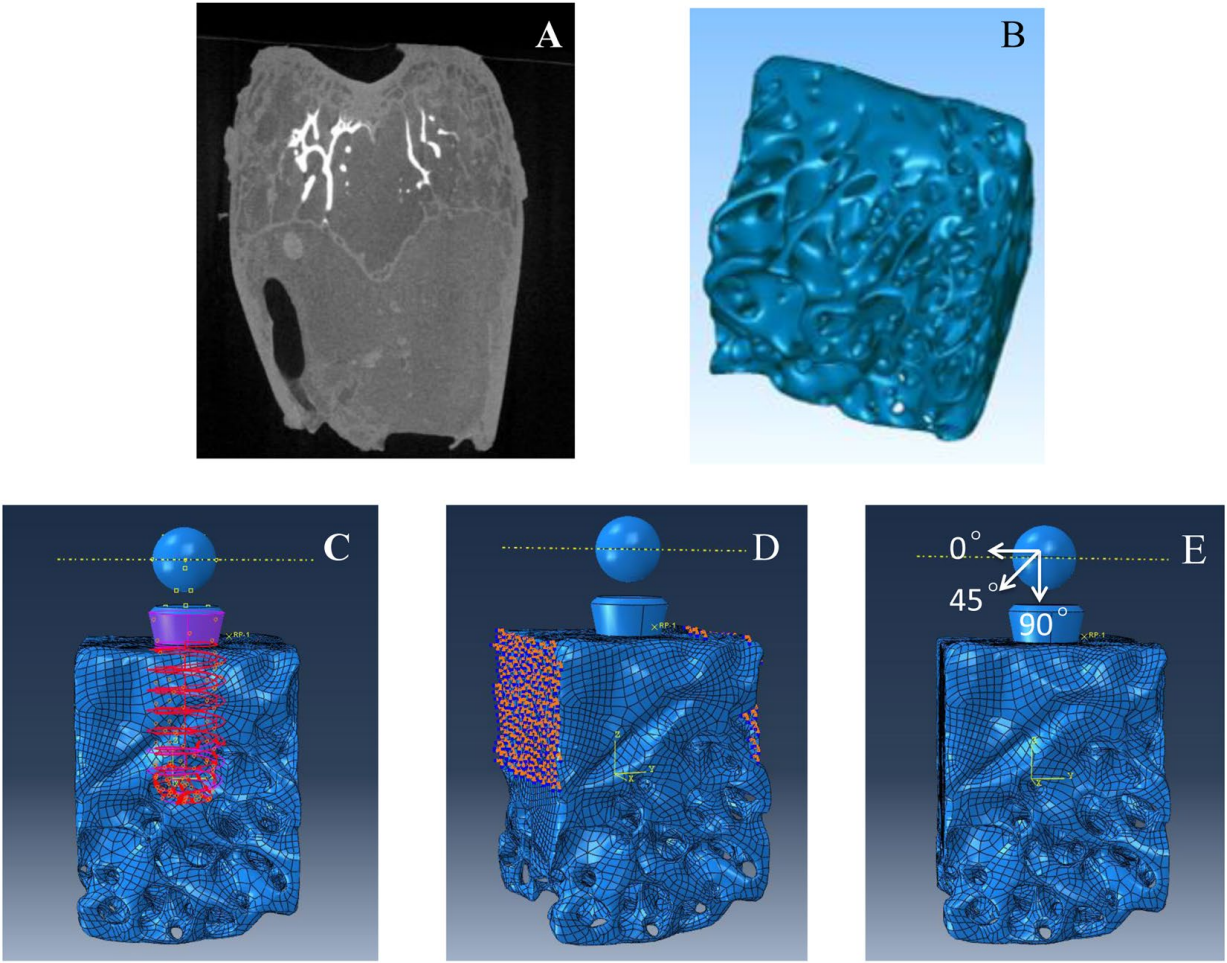
Three-dimensional FE model in Abaqus 6.13 software (Dassault System, Simulia Corp, USA). (**A**) Micro-CT images of femoral condyle of rabbit. (**B**) 3D solid structure of bone tissue. (**C**) Tie-constraint between the implant and bone tissue. (**D**) Boundary condition of the model. (**E**) Simulation model under impact loading at 0°, 45°, and 90° along the axis of the implant.

Micro-CT images of animals without implants were collected and imported into Mimics 15.0 (Materialise, Belgium) in DICOM format for 3D digital reconstruction (Fig. 3A). The grayscale threshold values of images were adjusted to obtain the sketch of cortical and cancellous bone. Images of each layer were edited to create closed contours. The contours in different layers were matched to reconstruct the 3D surface of the bone, as described previously^13,14^. Then the surface model was imported into Geomagic Studio 13.0 (Rain Drop, Triangle Park, NC) in STL format. The defective and deformed surfaces were repaired and flattened in Geomagic Studio to yield a shell structure of non-uniform rational B-spline (NURBS) curved surfaces^15^. Finally, the NURBS curved surfaces were imported into UGNX10.0 (Siemens PLM Software, Germany) in IGES format to obtain the solid microstructure^16,17^.

3D structure of implant was generated with UGNX10.0 software. Bone tissue established was overlapped with the implant in proper position where the implant was inserted in animal experiment. After that, the region of bone tissue overlapped by the implant was removed by subtraction of Boolean calculation to establish bone-implant solid structure^18^. Then, the structures were saved in. prt format for developing the FE model.

#### Establishment of a 3D FE microstructural model of implant and bone

The solid structure of bone (Fig. 3B) with implant was imported into Abaqus 6.13 (Dassault System, Simulia Corp, USA) software in. prt format. External bone with compact structure in thickness of 1.5 mm was selected as cortical bone, while bone with microstructure was considered to be cancellous bone. The material parameters used for cortical bone and cancellous bone are presented in Supplementary Table 1. The interface between bone and implant was defined as “tie-constraint” to simulate good osseointegration^19,20^ (Fig. 3C). Displacement on opposite sides of the bone in the model was limited to zero in all three directions (Fig. 3D).

In simulating impact load, a rigid body with diameter of 1.5 cm was generated, and the model was assembled to ensure that the rigid body was located above the implant, 2 mm away from the implant surface. The rigid body was loaded with initial velocity (impact velocity) to impact on the implant paralleling to the long axis of the implant according to the animal experiments. The contact between the rigid body and the implant surface was defined as frictionless, and the rigid body was forced to be removed immediately after contact with the implant surface. The model was meshed with a tetrahedral element (29,4325units), as reported by Li *et al*.^18^.

### Simulation analysis of bone damage under impact

In simulation of bone damage, a user material subroutine compiled by Fortran Language was used to judge the effectiveness of elements in the model according to the results of the dynamic mechanical test. The von Mises stress was used as an analysis criterion. When the von Mises stress of the model units reached the yield strength, these units were defined as ineffective and would be deleted, otherwise, they were retained and formed a steady-state model.

The damage characteristic of bone was investigated under different loading conditions. Analysis with load rates (4.0 m/s, 5.1 m/s, and 6.3 m/s) corresponded with animal experiments and at loading directions of 0°, 45°, 90° (along the axis of the implant) were performed respectively (Fig. 3E). Convergence tests with mesh refinements were performed. The von Mises stress in the bone was used for convergence monitoring, and a tolerance of 5% was employed.

### Statistical analysis

Data for BV/TV and Tb.Sp in Micro-CT analysis and data for BIC in histological analysis were presented as mean ± standard deviation. One-way ANOVA was performed with SPSS 19.0 (IBM, USA). Statistical significance was considered as P < 0.05.

## Results

### Evaluation of bone tissue surrounding the implant in animal test

#### Osseointegration after implantation

**Figure 4:**
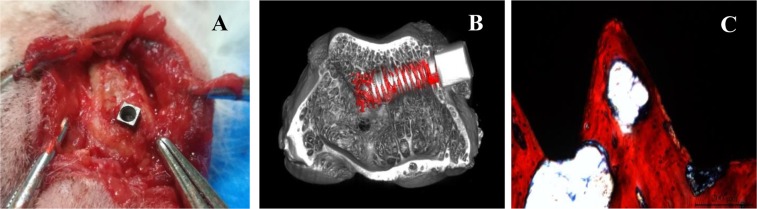
Bone formation for osseointegration after 3 months. (**A**) Healing of cortical bone. (**B**) Micro-CT images show good osseointegration in the area surrounding the implant. (**C**) VG staining shows favourable implant-bone contact.

Hard and soft tissue in the implantation area healed nicely without infection at 3 months postoperatively. The implant was combined with the cortical bone closely and fixed tightly. Slight cortical bone hyperplasia was observed in the area surrounding the implant (Fig. 4A).

Micro-CT images revealed that the implant was located at the distal femoral condyle. The screws and the bottom of the implant had been filled with cancellous bone consisting of thick and regularly arranged trabeculae (Fig. 4B).

The formation for osseointegration between the implant and bone was observed through VG staining. The implant’s screws were full of intact trabeculae. The implant combined with the bone tightly without fibrous tissue, and favorable osseointegration was observed at the implant-bone interface (Fig. 4C).

#### Damage in bone tissue after impact loading

**Figure 5:**
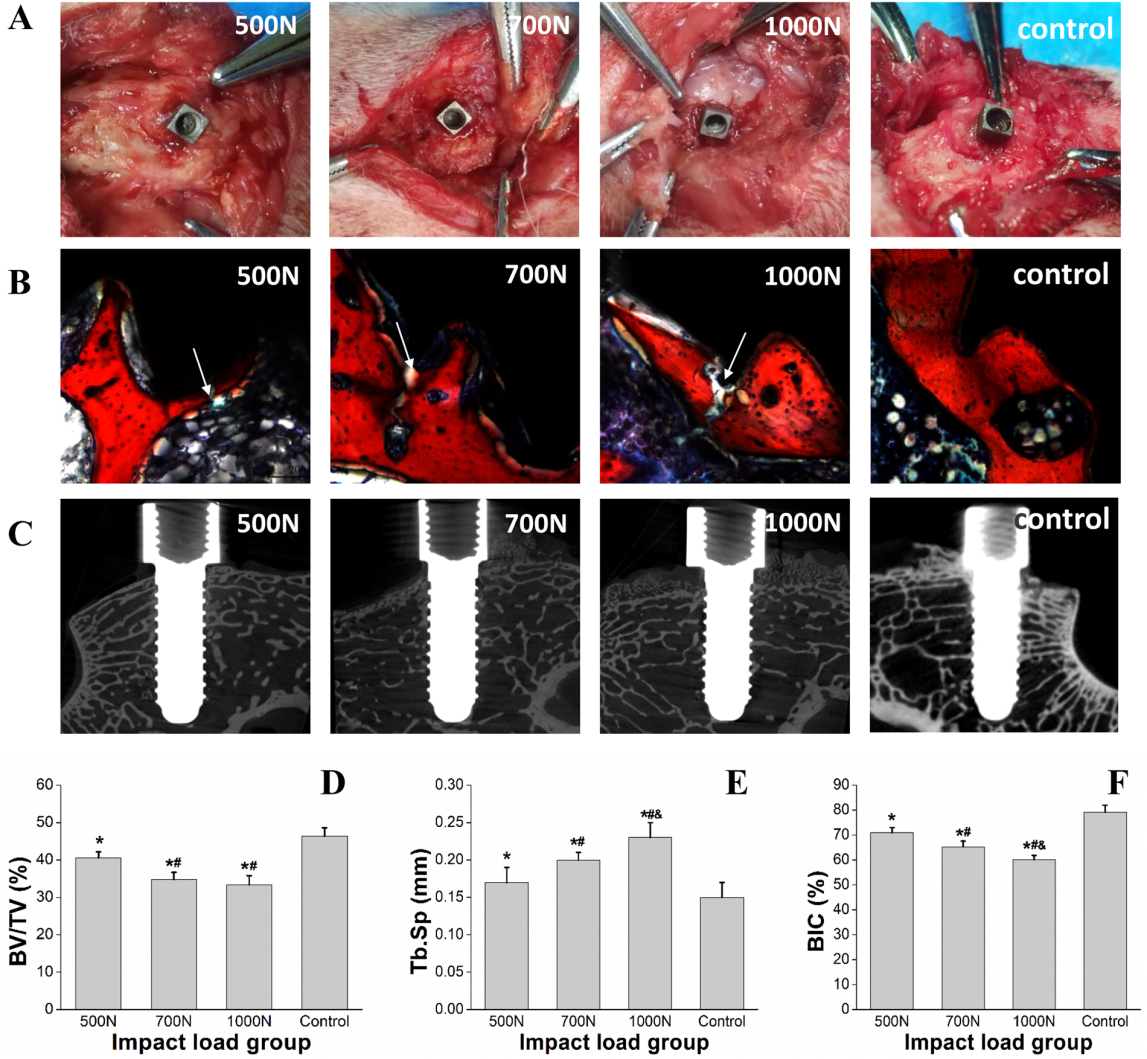
Bone damage caused by the impact in the area surrounding the implant. (**A**) Damage to cortical bone in the area surrounding the implant. (**B**) VG staining showing trabecular bone fracture. (**C**) Micro-CT images showing trabecular bone fracture. (**D**) Data for BV/TV in Micro-CT analysis are expressed as x ± SD (n = 6), and one-way ANOVA was performed with SPSS 19.0. *Indicate significant difference between the test groups and the control group with P < 0.05; ^#^indicate significant difference between the 700N, 1000N group and the 500 group with P < 0.05. (**E**) Data for Tb.Sp are expressed as x ± SD (n = 6), and one-way ANOVA was performed with SPSS 19.0. *Indicate significant difference between the test groups and the control group with P < 0.05; ^#^indicate significant difference between the 700N, 1000N group and the 500N group with P < 0.05; ^&^indicate significant difference between the 1000N group and 700N group with P < 0.05. (**F**) Data for BIC in histological analysis are expressed as x ± SD (n = 6) and one-way ANOVA was performed with SPSS 19.0. *Indicate significant difference between the test groups and the control group with P < 0.05; ^#^indicate significant difference between the 700N, 1000N group and the 500N group with P < 0.05; ^&^indicate significant difference between the 1000N group and 700N group with P < 0.05.

No obvious damage to cortical bone was found in the 500-N or 700-N groups, but bone fracture and depression were observed in the 1000-N impact group (Fig. 5A). In the experimental groups, histological examination (Fig. 5B) and Micro-CT images (Fig. 5C) revealed fractures in the trabecular bone surrounding the implant. Micro-CT analysis showed that BV/TV had decreased significantly in test groups (P < 0.05) (Fig. 5D), compared with the control group. Although no significant changes were observed in the 700-N and 1000-N group, the difference between the 500-N group and the other two test groups was significant (P < 0.05). Tb.Sp increased significantly in test groups compared with the control group (P < 0.05; Fig. 5E). The differences between test groups were also significant (P < 0.05). The histological analysis showed that the BIC in test groups decreased significantly compared with the control group (P < 0.05; Fig. 5F) and there were also significant differences between test groups (P < 0.05). t The results suggest that bone structure surrounding the implant is prone to be damaged under impact, and the degree of damage is related to the magnitude of the loading.

### Dynamic mechanical properties of the rabbit femur

**Figure 6:**
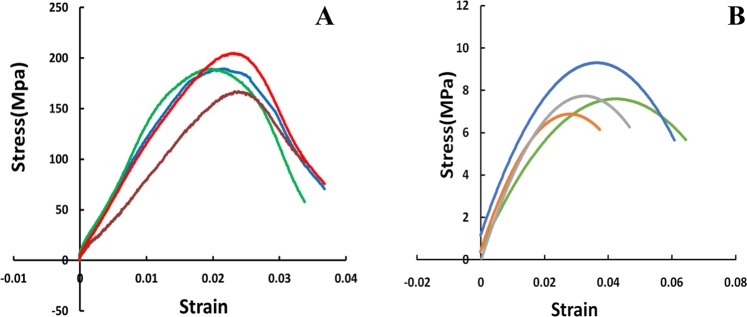
Dynamic stress-strain curve for rabbit femur bone tissue. (**A**) Cortical bone; (**B**) Cancellous bone.

The stress-strain curves for cancellous and cortical bone after impact loading are shown in Fig. 6. With an increase in stress, the specimen deformed without obvious damage, and the stress-strain curve changed almost linearly. When the stress reached a certain value, the specimen yielded, then the stress value decreased rapidly during plastic deformation. The yield strength of cortical bone (180 ± 10.5 MPa) was significantly greater than that of cancellous bone (8.9 ± 1.2 MPa) (Fig. 6A), but the plastic deformation of cancellous bone was superior to that of cortical bone (Fig. 6B).

### Simulation of bone damage surrounding the implant under impact loading

#### The bone damage process surrounding the implant under impact load

In the simulation of bone damage, the von Mises failure criterion was used in combination with VUMAT to evaluate the structural damage.

**Figure 7:**
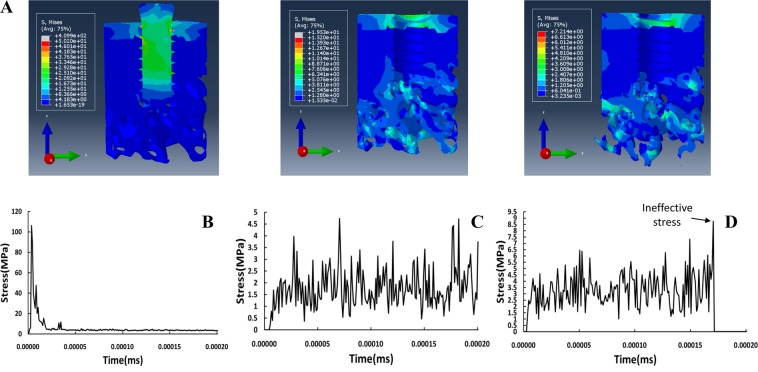
The response of bone tissue surrounding the implant after impact loading. All the figures presented were from Abaqus 6.13 software (Dassault System, Simulia Corp, USA). (**A**) The distribution of stress in bone tissue during impact loading and the subsequent prorogation of stress waves. (**B**) Stress-time curve for cortical bone under impact loading. (**C**) Stress-time curve for cancellous bone under vertical impact loading. (**D**) Stress-time curve for cancellous bone under impact loading at 45°.

Figure 7A shows that, after vertical loading, the stress transmitted from the implant to cortical bone increased rapidly, resulting in stress concentration. The stress-time curve for cortical bone in this area also confirmed this change (Fig. 7B). In contrast, stress in cancellous bone increased gradually after unloading. The stress-time curve for cancellous bone at the bottom of implant also show that stress changed over time (Fig. 7C,D). When the stress value reached the yield strength, the structure of cancellous bone was damaged and ineffective (Fig. 7D). The results of this simulation indicate that the impact load is transferred to cancellous bone in the form of stress waves, contributing to the fracture of trabecular bone and the failure of osseointegration. The areas of bone micro-damage highlighted by the simulation analysis were consistent with the results of the animal experiments described above (Fig. 5B,C), confirming the reliability of the numerical model.

#### The bone damage characteristic surrounding the implant under impact load

##### Effect of loading magnitude on bone damage

**Figure 8:**
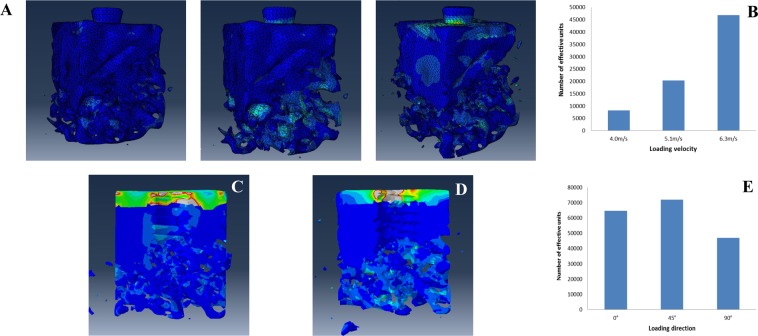
The relationship between bone damage and loading magnitude and loading direction. All the figures presented were from Abaqus 6.13 software (Dassault System, Simulia Corp, USA). (**A**) The effect of loading velocity (4.0 m/s;5.1 m/s;6.3 m/s) on damage. (**B**) The number of ineffective units in the model at a given loading velocity; (**C**) Horizontal impact loading. (**D**) Oblique impact loading at 45°. (**E**) The number of ineffective units in the model associated with a given loading direction.

Figure 8(A) shows the damage situation of bone at various vertical loading rates. The stress contour demonstrated that the degree of damage to trabecular bone at the bottom end of the implant and at the bone-implant interface increased with the increases of load magnitude. The number of ineffective units in the model increased accordingly Fig. 8(B). The extent of damage to cortical bone ranged from invisible bone defects to fracture. The results were consistent with those provided by micro-CT analysis of animal experiments (Fig. 5D–F).

##### Effect of loading direction on bone damage

Figure 8(C,D) shows that the situation of bone damage was closely related to the direction of impact loading. When the impact force was loaded in the horizontal direction, stress concentrated in cortical bone, but no obvious damage was found. Damage to trabecular bone was observed mainly at the bottom of the implant, and some of the trabecular bone fractures occurred at the bonding interface (Fig. 8C). When impact force was loaded obliquely at 45°, the stress value and the range of stress distribution increased. The bone-implant interface and contralateral cancellous bone showed clear signs of damage (Fig. 8D). This was also confirmed by the results that the number of ineffective units in the model of loading at 45° were more than that of loading at other directions (Fig. 8E). The stress-time curves demonstrated that the time when cancellous damage occurred at the bottom of the implant was after impact load (Fig. 7D). The results for vertical loading were presented above. All these results indicate that the location and extent of bone damage surrounding the implant may be affected by loading direction and subsequent propagation of stress waves that was proved by stress-time curve for cancellous bone at the bottom end of the implant loading at 45° (Figs. 7D and 8D).

## Discussion

The success of a dental implant depends on the maintenance of successful osseointegration and the support of good-quality bone^2,21^. In contrast to natural teeth, which are buffered with periodontium, dental implant transfers the force directly to surrounding alveolar bone under impact. This force will damage peri-implant bone and destroy osseointegration easily. Studying the process of impact damage and exploring the characteristics of bone damage will help in the clinical diagnosis and treatment of patients who have been subjected to impact. Therefore, in the present study, implants were inserted into the femoral condyles of New Zealand rabbits to establish the animal model. Implants with good osseointegration were loaded with impact forces. A 3D FE model was developed based on the animal experiment. This model was used to simulate the process of bone damage and investigate characteristics of damage in combination with data from dynamic mechanical experiments of the femur. The results of animal experiments as well as the simulation data showed that impact loading contributed to the development of bone damage due to stress concentration around the implant. The subsequent propagation of energy and stress waves may damage osseointegration and bone-implant interface^21^. The extent of damage to the bone tissue surrounding the implant ranged from invisible damage of trabecular bone structure and the failure of osseointegration, to fractures in the cortical bone and subsequent loosening of the implant. The bone damage condition was related to the magnitude and direction of the force applied. The results presented above indicate the value of a thorough examination and evaluation of damage to patient before treatment in clinics.

Studies reporting that impact loading results in bone damage typically focus on the femur or skull^22,23^. No previous report has investigated the response of alveolar bone surrounding the dental implant to impact. In order to investigate the process and characteristics of bone damage accurately, a numerical model of implant and bone tissues with microstructure should be established. New Zealand white rabbits are commonly used as an animal model for research on dental implants, as the femur’s cancellous bone and thin cortical bone have structures similar to those of alveolar bone. So, in this study, the femoral condyles of New Zealand rabbits were used to characterize bone structural damage in animal test. The data obtained were used to establish a numerical model in order to reveal the mechanism of alveolar bone damage surrounding dental implant under impact. When establishing the FE model, it was very difficult to reconstruct the 3D morphologies of the implant and bone tissue simultaneously because of the metallic implant artifacts in Micro-CT. To maximize the accuracy of presented simulation, the microstructure of bone tissue and the implant were established separately. The femoral condyles without implant were selected for scanning and rebuilding. The implant was established using UGNX10.0 software and was assembled with bone tissue in the model using Boolean subtraction^18^. The interface between the implant and the bone tissue was defined as “tie-constraint “to simulate bone formation for osseointegration^19,20^. Moreover, it was also very difficult to reconstruct trabecular microstructure. Grayscale threshold values were adjusted appropriately in micro CT images using Mimics 15.0 to obtain sketches of trabecular structure. Notably, some trabecular microstructures were neglected because the associated shape was too complicated or too small to be used for reconstruction. After comparison with the results of animal experiments, the 3D FE model presented, which reflected the microstructure of trabecular bone and implant, appears to be appropriate for use in analyzing bone damage surrounding the implant under impact.

The dynamic properties of rabbit femur were essential for simulating bone damage under impact force. The dynamic mechanical testing of the rabbit femur was conducted using a separate Hopkinson pressure bar. Use of a Hopkinson bar allowed us to adjust bullet mass and velocity at a specific strain rate to simulate the impact loading used for the animal experiments^24^. The yield strength of cortical bone and cancellous bone were obtained from the dynamic stress and strain curves which were determined from the results of the animal test based on one-dimensional stress wave theory^11,12^. Compared with the dynamic mechanical properties of bone tissue from other methods such as drop-hammer method, the stable and reproducible results could be gained by Hopkinson bar test and the dynamic stress and strain curve could also be constructed precisely^24^.

In simulating impact damage, the user subroutine based on the dynamic yield strength of cortical and cancellous bone was used to evaluate the effectiveness of an element in the model. When the stress in bone tissue reached the yield strength, this element would be found ineffective and deleted; otherwise, the analysis would be continued. The stress distribution and stress-time curve showed that the peak stress occurred immediately in cortical bone surrounding the implant after impact loading, and then the high stress decreased rapidly. In contrast, the stress in cancellous bone increased gradually, and the cancellous bone damage occurred until the stress reached yield strength (Fig. 7D). The stress-time curves of cancellous bone demonstrated that the peak stress appeared at different time-points compared with cortical bone (Fig. 7C,D). These findings suggest that impact load may be propagated and conducted as stress waves after unloading. Propagation and repeated reflection of the stress wave between the implant and the bone tissue is prone to destroy the trabecular structure and bring about the failure of osseointegration. Accordingly, in addition to the structural bone damage caused by the shock force in the loading direction, subsequent damage arose from impact waves in the bone tissue deserves more attention^25^. The data from animal experiments also demonstrated that trabecular bone fracture occurred at the bottom end of the implant along the direction of the loading, and the fracture and the failure of bonding interface occurred at the sites away from the implant likewise. Although the location of damage on the simulated map may differ from that predicted by the results of animal testing, the extent of damage to bone tissue agreed with results obtained by Micro-CT scan and data analysis. All these results indicate that the result of simulation is consistent with that of the animal experiment, and the numerical simulation model captured the damage process and characteristics of the bone tissue surrounding the implant during impact loading. These findings may be applied for further research on the remodeling of bone tissue induced by biting forces after impact damage.

During impact loading on an implant, the degree of bone damage is dependent on impact energy. The velocity of loading, an important ingredient of impact energy, is closely related to the severity of damage^26,27^. The present study showed that, when the implant suffered low-velocity impact, no obvious damage was found on the surface of cortical bone around the implant, despite the concentration of stress in this area. However, trabecular structure was fractured at the bottom of the implant under the action of stress waves. These findings suggest that thorough examination and evaluation should be performed to patients, even no obvious change is found in clinic. While, high-velocity impact damaged the cortical bone, resulting in fracture of the osseointegration interface and trabecular bone, thus compromising the stability of the implant. Under this condition, adjustment of occlusal forces or removal of the upper structure of dental implant should be considered according to the clinic examination and CT scan in order to promote reosseointegration.

The results also showed that the direction of impact loading was another factor affecting the bone damage. The propagation of stress waves changed with the direction of impact loading influencing the pattern of bone damage consequently^27,28^. When the impact force was vertically loaded, although the stress wave propagated and dispersed in the direction of loading, trabecular bone damage at the bottom of the implant and the failure of bonding interface had been alleviated by the cortical bone. In contrast, when impact force was obliquely loaded which is a common crash type in clinic, the stress wave propagated and reflected repeatedly between the implant and the bone tissue. Under this condition, trabecular bone damage and the failure of osseointegration would be aggravated correspondingly. Finally, when impact loading was in the horizontal direction, the propagation and reflection of the stress wave occurred mainly along this direction. The cancellous bone damage caused by shock load would be buffered with the cortical bone at the opposite side of the loading. Meanwhile, the cancellous bone damage caused by the stress wave at the implant-bone interface or at the bottom of the implant were also mediated. Therefore, the damage condition was worst when the impact force was loaded obliquely at 45°. These results were also proved by the number of ineffective element in the simulation under different loading conditions (Fig. 8E). The results of simulation analysis indicate that cortical bone with superior mechanical properties plays a very important role in absorbing and buffering the energy of the impact. Therefore, the damage condition of cortical bone and the stability of the implant must be evaluated to determine the treatment planning for the patients suffering from impact injury.

entalstability of the implant.

In conclusion, the empirical results obtained from our animal model, in combination with simulation data, show that the stress wave formed by an impact force can easily damage trabecular bone as well as osseointegration surrounding the implant. These changes may compromise the stability of the implant and the prognosis of the patient. The extent of the trauma caused by impact loading depends on the magnitude and direction of the impact force, suggesting that patients with loading damage should be comprehensively examined and evaluated.

## Supplementary Information

Supplementary Information.
