# Supplementary material for: Simulation analysis of impact damage to the bone tissue surrounding a dental implant
Source: Sci Rep. 2020 Apr 24;10:6927. doi: 10.1038/s41598-020-63666-5 (PMC7181623; doi:10.1038/s41598-020-63666-5)
Supplement: Supplementary file 1 — Supplementary Information. [file 41598_2020_63666_MOESM1_ESM.pdf]

Simulation analysis of impact damage to the bone tissue surrounding a dental implant

Xinyang Ma[1, 2], Xiaoou Diao[1], Zhirui Li[1], Haitao Xin[1], Tao Suo[3], Bing Hou[3], Zhongbin Tang[3], Yulu Wu[1], Fan Feng[1], Huiwen Luo[1]

1 State Key Laboratory of Military Stomatology & National Clinical Research Center for Oral Diseases & Shaanxi Key Laboratory of Stomatology, Department of Prosthodontics, School of Stomatology, The Fourth Military Medical University, Xi'an, 710032, China.

2 School of Stomatology, XI'AN Medical Univercity, Xi'an 710021, China.

3 School of Aeronautics, Northwestern Polytechnical University, Xi'an 710072, China

Xinyang Ma and Xiaoou Diao contributed equally to this work.

Correspondence and requests for materials should be addressed to Haitao Xin (email: xhthmj@fmmu.edu.cn)

Supplementary Table 1 The parameters of material

Material	Young's modulus（GPa）	Poissons'ratio	Density(g/cm^3)
Implant	114	0.3	4.51
Cortical bone	18.0	0.3	1.74
Trabecular bone	0.76	0.3	0.9
Rigid body	120	0.3	7.9
